# Supplementary material for: Epidemiology of disorders associated with tall stature in childhood: A 20-year birth cohort study
Source: PLoS One. 2025 Apr 15;20(4):e0321798. doi: 10.1371/journal.pone.0321798 (PMC11999151; doi:10.1371/journal.pone.0321798)
Supplement: S1 Table — (PDF) [file pone.0321798.s001.pdf]

Supporting information

S1 Table. Congenital malformation syndromes involving early overgrowth: specific name and frequencies during the follow-up.

Name of congenital overgrowth syndrome	Frequency, n (%)
Beckwith–Wiedemann syndrome	
Girls	38 (38.4)
Boys	43 (42.6)
Sotos syndrome	
Girls	41 (41.4)
Boys	40 (39.6)
Other congenital overgrowth syndrome	
Girls	20 (20.2)
Boys	18 (17.8)
**Total**	
Girls	99 (100.0)
Boys	101 (100.0)
