# Supplementary material for: Epidemiology of disorders associated with tall stature in childhood: A 20-year birth cohort study
Source: PLoS One. 2025 Apr 15;20(4):e0321798. doi: 10.1371/journal.pone.0321798 (PMC11999151; doi:10.1371/journal.pone.0321798)
Supplement: Attachment — Submitted filename: Response to reviewers.pdf [file pone.0321798.s002.pdf]

Response to Reviewers

Journal Requirements:
When submitting your revision, we need you to address these additional requirements.

1. Please ensure that your manuscript meets PLOS ONE's style requirements, including those for file naming.

> **AU:** We have now checked that the manuscript meets PLOS One's style requirements.

2. We note that you have indicated that there are restrictions to data sharing for this study. PLOS only allows data to be available upon request if there are legal or ethical restrictions on sharing data publicly. For more information on unacceptable data access restrictions, please see http://journals.plos.org/plosone/s/data-availability#loc-unacceptable-data-access-restrictions. Before we proceed with your manuscript, please address the following prompts: a) If there are ethical or legal restrictions on sharing a de-identified data set, please explain them in detail (e.g., data contain potentially identifying or sensitive patient information, data are owned by a third-party organization, etc.) and who has imposed them (e.g., a Research Ethics Committee or Institutional Review Board, etc.). Please also provide contact information for a data access committee, ethics committee, or other institutional body to which data requests may be sent.

> **AU:** Thank you for pointing this out. The permissions obtained from the maintainers of the data (THL, Statistics Finland, and Social Insurance Institution), as required by the Finnish legislation, do not allow the sharing of data. Some of the conditions associated with tall stature are extremely rare and the data may contain potentially identifying or sensitive patient material. However, other researchers can request similar register data from the Finnish Social and Health Data Permit Authority (Findata; https://findata.fi/en).
>
> We have now rewritten the data-sharing statement in the revised manuscript.

3. Please include captions for your Supporting Information files at the end of your manuscript, and update any in-text citations to match accordingly.

> **AU:** Caption for Supporting Information is now included at the end of the manuscript and in-text citations match accordingly.

4. Please review your reference list to ensure that it is complete and correct. If you have cited papers that have been retracted, please include the rationale for doing so in the manuscript text, or remove these references and replace them with relevant current references. Any changes to the reference list should be mentioned in the rebuttal letter that accompanies your revised manuscript. If you need to cite a retracted article, indicate the article's retracted status in the References list and also include a citation and full reference for the retraction notice.

> **AU:** We have now double-checked that we do not cite any papers that have been retracted (until November 15, 2024). We have updated reference numbers 36 and 37 (in the former reference list) to refer to the original studies. A few references became useless after the revision and shortening of discussion and have been removed. The changes can be seen in the "Manuscript with track changes", which includes the former and current reference lists.

Additional Editor Comments:

Dear Authors-
Please revise according to the reviewers' comments.

In particular, the more specificity of the disorders that can be provided, the more value the manuscript will have for readers.
Please try to be as specific as possible, do no aggregate disorders. The reviewer is requesting disaggregation.

> **AU:** Thank you for these comments. We have now revised the manuscript based on the reviewers' comments. To be as specific as possible, we follow the ESPE (European Society of Paediatric Endocrinology) classification of paediatric disorders associated with tall stature (Wit JM, Ranke MB, Kelnar CJH. ESPE classification of paediatric endocrine diagnoses. Horm Res. 2007;68(2):8-13). Each disorder is managed separately, and girls and boys are presented as their own groups. Likewise in ESPE classification, congenital overgrowth syndromes are handled as a single disease entity since they strongly overlap each other and they are also very rare. However, information on the exact frequencies of congenital overgrowth syndromes is given in the Supporting information at the end of the manuscript. In some cases, disorders are grouped as primary (congenital), secondary (acquired) or idiopathic tall stature. This division is important and it also has significance in clinical work.

Reviewer #1: The manuscript is a retrospective cohort study that investigated the cumulative incidence of primary and secondary disorders associated with tall stature in Finland over a 20-year period, from 1998 to 2017, among children up to 18 years of age. The findings suggest that some disorders remain undiagnosed. Special focus is given to Marfan syndrome, congenital overgrowth syndromes, Klinefelter syndrome, and central precocious puberty, which presented higher cumulative incidences.

The research appears to be well-conducted; however, the paper is somewhat confusing in regards to the diseases investigated. Specifically, there is inconsistency in the syndromes discussed across the abstract, introduction, and methods sections. Not all of the disorders are mentioned in each section, making it difficult for the reader to follow.

> **AU**: Thank you for pointing this out. Throughout the manuscript we stick to the European Society of Pediatric Endocrinology (ESPE) classification of tall stature disorders (Wit JM et al, 2007) and to the basic division of these conditions into primary and secondary disorders. Many of those conditions are rare. To improve the manuscript's readability, we have now limited the number of disorders presented in the Abstract to a few examples of the most common primary and secondary conditions associated with tall stature. However, in the other parts of the manuscript (Introduction, Methods and Results), more comprehensive and detailed disease-specific data are presented to provide a thorough outline of various conditions behind tall stature in childhood.

Minor issues:
The background section of the abstract could benefit from a clearer statement of the research aim.

> **AU:** Thank you for the comment. We have revised the abstract so that the research aim is more clearly stated: "This retrospective population-based cohort study aims to specify the epidemiological data that are needed to develop better diagnostic practices."

In the introduction, adding more epidemiological information about the disorders investigated would be helpful, either individually or grouped as primary and secondary growth disorders if literature is scarce. Including global prevalence or incidence rates, especially in countries with similar characteristics to Finland, would strengthen this section. As it stands, it feels more like a list of syndrome definitions.

> **AU:** Thank you for the suggestion. We have now included general incidences of several disorders (Marfan syndrome, Klinefelter syndrome, XYY syndrome, and triple X syndrome) in the introduction. We also believe it is helpful for readers. However, it is important to remember that those values are not directly comparable with the age- and sex-specific cumulative incidences found in the present study. We did not find other studies reporting age- and sex-specific cumulative incidences for the selected disorders.

Make the importance of the research clearer and highlight why such rare conditions should be investigated.

> **AU:** Thank you for your suggestion. We agree that many of the chosen conditions are rare and it may raise questions regarding their value as research targets. We have now added a short phrase to the abstract and we believe it makes the research aim and importance clearer: "This retrospective population-based birth cohort study aims to specify the epidemiological data that is needed to develop better diagnostic practices."
>
> However, the epidemiology of disorders associated with tall stature is so poorly characterized that it already gives us a basis to investigate it. Rare disorders may have various lifelong consequences for the patient and his/her close relatives and should therefore not be considered insignificant. Furthermore, this kind of long-term nationwide cohort study is a reliable and often the only way to search the epidemiology of rare disorders. We try to underline the importance of the study also at the end of the introduction: "Such data are needed to develop evidence-based growth-screening programs and better diagnostic practices of disorders associated with tall stature in paediatric health care worldwide. Timely diagnosis of underlying conditions is important to prevent permanent effects on physical health and adult height and to support children's development."

In line 76, the term "essential disorders" could be clarified by listing the specific disorders the paper focuses on, helping the reader to know what to expect in the methods and results sections. Furthermore, in lines 93-99, not all of the investigated disorders are mentioned.

> **AU:** Thank you for pointing this out. We have replaced the word "essential disorders" with "several primary and secondary disorders" that we have listed in the previous paragraphs.
>
> We now list all the specific disorders focused on in the Methods (lines 96–99). Now it reads as follows: "The following primary or secondary disorders associated with tall stature were selected for analyses: <u>KS</u> (boys; ICD-10 codes Q98.0, Q98.1, Q98.4), <u>XYY</u> (boys; Q98.5), <u>triple X</u> (girls; Q97.0), <u>FXS</u> (Q99.2), <u>MFS</u> (Q87.4), <u>COS</u> (Beckwith–Wiedemann Q87.30, Sotos Q87.31, other Q87.38), <u>CAH</u> (E25.00, E25.01), <u>hyperthyroidism</u> (E05.0–E05.2), and <u>CPP</u> (E22.80 and E30.1). Idiopathic stall stature (<u>ITS</u>; familial or constitutional and non-familial: E34.40) was also included."

The discussion section is overly long and could benefit from summarization. The authors could prioritize key findings at the beginning, dedicating more space to discussing them in relation to existing literature. Less significant findings can follow, discussed in a more concise manner.

> **AU:** Thank you for the suggestion. The discussion is now considerably shorter and rewritten accordingly. We hope that it is now more readable and concise.

When comparing this manuscript's results with those from other studies, provide more context about those studies (e.g., location, study period, etc.), so that the reader can assess how comparable the results are.

> **AU:** Thank you for your comment. We have now added more context to the referred studies in the discussion section.

Reviewer #2: Is the manuscript technically sound, and do the data support the conclusions?

I believe the manuscript is technically sound and the data support the conclusions because of the following:
- The problem, study aim, and possible benefits from this research is well defined and stated in the introduction section.
- Results support the suggestion that during early childhood, growth screening should take place among patients with MFS and congenital overgrowth syndromes, with the addition of KS and CPP in later years.

> **AU:** Thank you for the positive feedback.

Has the statistical analysis been performed appropriately and rigorously?

- Aalen Johansen cumulative incidence function is appropriate. However, it is important explain why the presentation of age groups in table 1 and 2 is important. Please explain the rationality behind age categories and why it is different for table 1 and 2.

> **AU:** In Table 1, the age groups 0, 2, 6, 10, 14, and 18 years are selected due to their clinical representativeness based on the biology of growth and are also common growth check-up points in Finnish health care. The regulation of linear growth in infancy (from 0 to 2 years) switches to the childhood growth phase which is ongoing at 6 years (preschool age). The age points 0, 2, and 6 years provide insight into the timely diagnosis of primary conditions. Thereafter, at 10 and 14 years (the school health care checks), the acquired conditions are increasing. The final age point, 18 years, gives us a rough estimate of the cumulative incidence of the disorder near adulthood. We have now added information on the selection of age points in the subtitle of Table 1.
>
> Precocious puberty is presented separately in Table 2 for technical reasons because the relevant age points differ from those used in Table 1. By definition, puberty is precocious if it is observed before age 8 in girls and before age 9 in boys. Therefore, these age points are used in Table 2. Data on the definition is now included in the title of Table 2.

- I don't see the graphs of the functions in the manuscript although I see title for Figure 1 and 2. I believe it may be technical problem.

> **AU:** We are sorry that you were unable to see the figures since we believe they are very demonstrative when comparing different disorders and sexes. We had, however, uploaded

the figures in the required format. The pdf version of the submission we received from the submission system displayed the figures correctly, so we had no reason to believe that there could be (technical) problems with them.

Overall, this is an interesting research. Please update the methods and results section acccordingly.

AU: Thank you. We have now updated the methods and result section as suggested.
