# Supplementary material for: Epidemiology of disorders associated with tall stature in childhood: A 20-year birth cohort study
Source: PLoS One. 2025 Apr 15;20(4):e0321798. doi: 10.1371/journal.pone.0321798 (PMC11999151; doi:10.1371/journal.pone.0321798)
Supplement: Attachment — Submitted filename: Response_to_Reviewers_auresp_2.pdf [file pone.0321798.s003.pdf]

Response to Reviewers

Additional Editor Comments:

1. The authors mention in the statistical analysis section that they gathered information on medication purchase - what was the rationale for this, and why was this not included as a study objective? If it is just for descriptive purposes, please clarify in the text (earlier in the methods section - and include the same reference as in the 2022 paper if relevant). Also, results are provided for medications but this is not mentioned in the discussion.

AU: Thank you for pointing this out. As you thought, the information on medication purchases is mostly for descriptive purposes, and thus, we did not consider it as our study objective.

We have now clarified the text in the method section as follows: “We also checked the purchases of medications used regularly for KS (testosterone, ATC code G03BA03), CPP (leuprorelin, L02AE02), and hyperthyroidism (antithyroid preparations, H03B) to get a crude estimate of the number of children using the specific medication.”

We also have some discussion on medication purchases (Manuscript lines 230–231, 272–274, and 287–290).

2. Given the 20 year study period, did the authors investigate whether results differed by time - perhaps in 5-year increments or is this not relevant?

AU: We thought about this during the early phase of the study. We also calculated incidence rates and incidence rate ratios of two different birth cohorts (e.g. birth year 1998–2007 versus birth year 2008–2017) with varying times of follow-up (e.g. from birth until the maximum of 4 years). The results revealed slightly more diagnoses of Marfan syndrome and congenital overgrowth syndromes in girls between different birth cohorts, but otherwise we did not find any significant differences. We decided not to include these results in the final paper. Although the study cohort of 1.14 million children is large, the number of diagnosed children is small due to the fact that these conditions are relatively rare. Further separation of boys and girls and comparison between different birth cohorts make the number of diagnosed children even smaller. Consequently, the cumulative incidence function from birth until the maximum of 18 years is statistically the most appropriate way to express the results.

On behalf of the Academic Editor, we received the message from the PLOS One Publications Assistant that we could disregard Comment #2. However, we decided not to remove the answer because we had already written it.

3. Regarding the national health registers, I don't have experience with these so am wondering if there is any instance where there would be discrepancies between registers? Or are individuals only in one register?

AU: Thank you for this interesting question. The national health registers used in the present study to search diagnoses were the Medical Birth Register (MBR) and the Care Register for Health Care (CRHC). The MBR forms the basis of the study because it includes all children born in Finland and their diagnoses during the first week of life. Since the exact time of diagnosis during the first week of life is not reported, we decided to set it as 0 years. After the first week of life, diagnostic data are recorded only in the CRHC. However, the CRHC also includes the diagnoses made during the first week of life, but there may be minor discrepancies in the time of diagnosis between the MBR and CRHC. This is mainly for technical reasons.

The CRHC also includes diagnoses of children who are not born in Finland. They were not included in the study because of the absence of diagnostic information at birth.

4. Is there a reference for the Aalen-Johnson estimator? I assume this estimator generates the 95% confidence intervals for the estimates? Please clarify this in the text.

AU: We now offer a reference concerning the Aalen-Johansen estimator (Coemans M, Verbeke G, Döhler B, Süsal C, Naesens M. Bias by censoring for competing events in survival analysis. BMJ. 2022 Sep 13;378:e071349. doi: 10.1136/bmj-2022-071349. PMID: 36100269).

Aalen-Johansen method estimates the cumulative incidence of the event of interest (accounting for competing events), and results are provided with the 95% confidence intervals. We have added 95% confidence intervals in Table 1 and Table 2.

5. Overall, I found that the text in the results section repeats the information in the Tables. Consider revising so the text highlights key findings without reiterating exact values that are presented in the tables. The 95% confidence intervals should be in the tables. I would also suggest including the interquartile range for the median age at diagnosis.

AU: Thank you for the comment. We have revised the results section accordingly. The 95% confidence intervals and interquartile ranges (from the 25th to 75th percentiles) have been added in Table 1 and Table 2.

6. The references need reformatting to conform to PLOS One guidelines (e.g., in reference 4, "[Internet]" is not needed and neither is "Available from". The doi should be in the format: doi: 10.1136/archdischild-2013-304830 - please check formatting of all references.

AU: References are now formatted according to PLOS One guidelines.

7. The 2022 study of this same cohort should be mentioned earlier in the paper than the discussion. In particular, it should be cited in the methods section especially since the approach was similar between studies. I note that the 2022 paper included a hypothesis whereas this paper does not. In addition, the 2022 paper mentions that 92% of children in the study population were of Finnish background - this should be included in the current paper as well. Similarly, the 2022 paper notes that data on growth measurements were not available.

AU: Thank you for the comments. We have made the revisions as suggested.
